# Psychological Distress Trajectories of Parents of Children With Developmental Disabilities Participating in a Parenting Intervention

**DOI:** 10.1111/jir.70037

**Published:** 2025-09-11

**Authors:** Emma Scripps, Paul Thompson, Peter E. Langdon, Richard P. Hastings, Bruce J. Tonge, Stewart L. Einfeld, Matthew R. Sanders, Kate Sofronoff, Kylie M. Gray

**Affiliations:** ^1^ Centre for Research in Intellectual and Developmental Disabilities (CIDD) University of Warwick Coventry UK; ^2^ Intellectual Disabilities Research Institute (IDRIS) University of Birmingham Birmingham UK; ^3^ Herefordshire and Worcestershire Health and Care NHS Trust Worcester UK; ^4^ Birmingham Community Healthcare NHS Foundation Trust Birmingham UK; ^5^ Department of Psychiatry, School of Clinical Sciences at Monash Health Monash University Clayton Australia; ^6^ Brain and Mind Centre University of Sydney Camperdown New South Wales Australia; ^7^ Parenting and Family Support Centre, School of Psychology University of Queensland Brisbane Queensland Australia

**Keywords:** developmental disability, intellectual disability, interventions, parents, psychological distress

## Abstract

**Background:**

Parents of children with developmental disabilities report higher levels of psychological distress. Parenting interventions may reduce parental psychological distress. Our aim was to investigate the psychological distress trajectories of parents receiving a parenting intervention.

**Method:**

Data were drawn from a state‐wide trial of Stepping Stones Triple P (SSTP) in Queensland and Victoria, Australia (*n* = 365 parents). Growth mixture modelling was used to describe psychological distress trajectories of parents of children with developmental disabilities and examine associations with the level of SSTP intervention received, child characteristics and financial hardship.

**Results:**

Three groups of parents/caregivers were identified, two of which presented a significant decline in psychological distress over time and one group presented no significant change. Additionally, higher child behavioural and emotional problems and lower adaptive skills were associated with poorer parent psychological distress over time. Level 4 of the SSTP intervention was also associated with steeper declines in psychological distress over time.

**Conclusions:**

Over a period of 18 months, with the implementation of the SSTP, parents' psychological distress tended to improve. Distinct groups of parents were identified based on different psychological distress trajectories. Findings indicate the significant role of child behavioural and emotional problems, children's adaptive behaviour and the level of SSTP in parent psychological distress trajectories.

## 1 Introduction

Developmental disabilities such as intellectual disability emerge during the developmental period of a child's life and are characterized by lifelong cognitive and adaptive impairments (World Health Organisation 2018). International evidence suggests a robust association between having a child with a developmental disability and higher levels of parental psychological distress (Arvidsdotter et al. 2016; Dykens et al. 2014; Hoyle et al. 2021; Scherer et al. 2019). Psychological distress is defined as a state of emotional suffering, expressed through traits of depression and anxiety, exhibited in response to a stressor (Drapeau et al. 2012).

Evidence has established an association between parent psychological distress and negative parental behaviour and engagement (Aunos et al. 2008; Dix and Meunier 2009) and subsequent negative outcomes for children (Galbally and Lewis 2017). Parenting interventions aim to improve parenting behaviour and engagement to subsequently improve children's outcomes and have been found to be successful in doing so (Ruane and Carr 2019; Sanders et al. 2014). However, our understanding of the influence of parenting interventions on parents' psychological distress over time is limited.

Theoretical frameworks are designed to explicitly inform interventions; the Pragmatic Integration of Theories framework highlights that parents exist within a broader structure, which influences how individuals adjust to life as a parent of a child with a developmental disability (Glidden et al. 2021). Factors that influence parental psychological distress exist within different systems. At the most immediate level, the parent–child dyadic system exists, whereby child characteristics have a direct influence on parent behaviour and adjustment. Supporting evidence has highlighted relationships between parental psychological distress and characteristics of children with developmental disabilities, including behavioural and emotional problems or mental disorders (Gray et al. 2011; Irazábal et al. 2012; Phetrasuwan and Shandor Miles 2009), adaptive skills (Webster et al. 2008; White and Hastings 2004), diagnosis of autism (Hayes and Watson 2013; Padden and James 2017) and age (D. Gray 2002; Tehee et al. 2009). Factors in the wider society are also influential but less immediately so; for example, the experience of financial hardship has been associated with poorer well‐being for parents of children with developmental disabilities (Emerson et al. 2006; Glidden et al. 2021).

The Pragmatic Integration of Theories suggests that parenting interventions should target parenting and parent–child relationships, with the aim to reduce children's behavioural and emotional problems, which will subsequently improve parent psychological adjustment (Glidden et al. 2021). However, theories are not often explicit about how interventions may influence parental psychological distress.

Stepping Stones Triple P (SSTP) is a behavioural family intervention, based on social learning principles, for parents of children with disabilities and aims to support parents to manage their child's behaviour (Sanders 1999). As a part of the intervention logic, the developers suggested that, as children's behavioural and emotional problems are predictors of parental distress, accessing behaviour change strategies and building more positive relationships with their child will directly reduce parental psychological distress (Plant and Sanders 2007). Randomized control studies of SSTP have reported no significant differences in levels of parental psychological distress post‐intervention (Plant and Sanders 2007; Roux et al. 2013; Sofronoff et al. 2011). However, SSTP has been found to reduce parenting stress 6 months after participation (Schrott et al. 2019) and reduce levels of depression 12 months later (Shapiro et al. 2014). Wider literature concerning parental distress trajectories during the implementation of parenting interventions is limited but has largely found a reduction in parenting distress (Chang and Fine 2007; Landsem et al. 2014). Therefore, there are discrepancies in the literature regarding the relationship between parenting interventions and parent psychological distress.

This study investigated parental psychological distress trajectories throughout a community‐based implementation of the SSTP intervention. This study aimed to examine the psychological distress trajectories of parents from pre‐intervention to 12 months post‐intervention, to understand the relationship between SSTP delivery and parental psychological distress. The second aim of this study was to explore associations between child behavioural and emotional problems, child adaptive skills, autism diagnosis, child age, financial hardship, the level of SSTP intervention received and parental psychological distress trajectories.

## 2 Methods

Data were drawn from the Mental Health of Young People with Developmental Disabilities (MHYPeDD) study, a nonrandomized evaluation of the implementation of SSTP in Australia between 2012 and 2017. Participant consent obtained for the original study covers additional research such as that reported here. This secondary data analysis was preregistered: osf.io/zcs3v.

### 2.1 Participants

In terms of inclusion criteria, parents/caregivers had to

1. have a child between the ages of 2 and 10 years.
2. live in the Australian state of interest (Victoria or Queensland).
3. have a child with a diagnosed intellectual disability (≥ 6 years) or a developmental disability (≤ 5 years) confirmed through provision of reports to the research team or standardized assessments.
4. have the necessary language skills to complete the questionnaires or have access to support to enable them to do so.

Data from 365 parents and caregivers of children between the ages of 2 and 10 years with a confirmed intellectual disability or developmental disability were included in the current study. Table 1 displays parent/caregiver and child demographic information.

**TABLE 1:** Parent/caregiver and child demographics.

Variables	*N*	Per cent
**Relation to child**		
Biological/adoptive/foster/step mother	324	88.8
Biological/adoptive/foster father	32	8.8
Grandparent	7	1.9
Other	2	0.5
**Marital status**		
Married	260	71.2
Cohabiting	40	11
Divorced/separated/single/widowed	59	16.2
Other	6	1.6
**Employment status**		
Employed	175	47.9
Unemployed	178	48.8
Full‐time education	9	2.5
**Education**		
Degree level and above	183	50.1
Below degree level	180	49.3
**State of residence**		
Queensland	101	27.7
Victoria	264	72.3
**Financial hardship**		
Yes	156	42.7
No	204	55.9
**Child gender**		
Male	257	70.4
Female	108	29.6
**Child age (years)**		Mean (SD)
	365	6.07 (2.28)
**Autism diagnosis (parent/caregiver reported)**		
Yes	230	63
No	135	37
**Vineland adaptive behaviour composite**		Mean (SD)
	365	63.55 (11.3)

### 2.2 Intervention

The MHYPeDD team provided SSTP training to 280 professionals who worked with children with intellectual and developmental disabilities in Victoria and Queensland. SSTP consists of five levels that increase in intensity; the MHYPeDD study evaluated Levels 2, 3 and 4. Level 2 (Selected SSTP) involves three seminars providing advice concerning minor child behaviour problems (Sanders et al. 2004). Level 3 (Primary Care SSTP) involves four, 15‐ to 30‐min, individual, narrow‐focus training sessions, with the focus determined by the parent/caregiver. Level 4 (Standard SSTP) has a broader focus with delivery possible in a variety of formats; the group sessions involve six, two and a half hour sessions and three individual telephone sessions (Sanders et al. 2004).

The majority of parents/caregivers participated in Level 4 (*n* = 268), 32 parents/caregivers participated in Level 3, and 65 parents/caregivers participated in Level 2. There were three assessment time points in this study: pre‐intervention (baseline), post‐intervention (3 months post‐baseline) and a 12‐month follow‐up. In terms of retention rate, at 3 months post‐intervention, the sample size declined from 365 to 291 (79.7%); this rose to 307 (84.1%) at the 12‐month follow‐up.

### 2.3 Measures

#### 2.3.1 Psychological Distress

The Depression, Anxiety and Stress Scales (DASS‐21) were used to assess parents'/caregivers' psychological distress (Henry and Crawford 2005; Lovibond and Lovibond 1995). This 21‐item self‐report questionnaire is composed of seven statements per subscale (depression, anxiety and stress). Statements are rated on a 4‐point Likert scale ranging from 0 (*did not apply at all*) to 3 (*applied very much or most of the time*). To generate a total measure for psychological distress, summed numbers in each subscale are multiplied by 2 to enable direct comparisons to the longer version of the DASS (DASS‐42) (Lovibond and Lovibond 1995). Subscale scores are then summed together; thus, total DASS‐21 scores range from 0 to 126 (Beaufort et al. 2017).

The DASS‐21 has good convergent and discriminant validity when compared with other anxiety and depression measures (Henry and Crawford 2005). The DASS‐21 has also yielded moderate to high rates of internal consistency (Osman et al. 2012). This shortened version of the DASS lessens the demand on participants, yet maintains adequate reliability (Henry and Crawford 2005). Scores from all three time points were considered in this study. The DASS‐21 across the three time points was found to have excellent internal consistency (*α* > 0.9).

#### 2.3.2 Child Behaviour and Emotional Problems

Child behaviour and emotional problems were assessed using the Developmental Behaviour Checklist Parent Form (DBC2‐P) (Gray et al. 2018). This 96 item measure assesses the behavioural and emotional problems of individuals aged 4–18 years with intellectual and developmental disabilities. All questions are rated on a 3‐point Likert scale of 0 to indicate *not true as far as you know* to 2 *very true or often true*. A version of the DBC2‐P (Gray et al. 2018) was adapted for parents/caregivers of children under 4 years (DBC‐U4) to ensure questions were age appropriate. To determine an overall score, all items were summed together and divided by the number of items contributing to that score to generate a Mean Item Score (MIS); benefits of using the MIS include a heightened tolerance for missing data (Taffe et al. 2008). The range for this score is between 0 and 2. The DBC has been found to have good test–retest reliability and high internal consistency with samples of children with intellectual and developmental disabilities (Gray et al. 2018). DBC MIS scores from all three time points were included in this analysis.

#### 2.3.3 Children's Adaptive Behaviour

Children's adaptive behaviour was assessed using the Vineland Adaptive Behavioural Scale‐Parent/Caregiver Self Report Form (VABS‐II); this measure evaluates adaptive behaviours of children and young people up to 18 years (Sparrow et al. 2005). Items are rated on a scale of 0 *never performed* to 2 *behaviour is usually or habitually performed*. Standard scores are calculated for the three domains: communication, daily living skills and socialization, and an adaptive behaviour composite standard score is also generated. The adaptive behaviour composite has been reported to have high split‐half reliability and convergent validity with other measures of adaptive behaviour (Sparrow 2011; Sparrow et al. 2005). The VABS‐ Parent/Caregiver Self Report Form was completed by parents/caregivers pre‐intervention.

#### 2.3.4 Financial Hardship

To measure financial hardship, participants were asked to report how difficult it would be for them to raise $2000 (AUD) in 1 week. Responses included they (1) *could easily raise the money*, (2) *could raise the money but this would involve sacrifice*, (3) *would have to do something drastic to raise the funds* or (4) *could not raise the money*. Responses were subsequently dichotomized into 0 (responses 1 and 2 indicating no financial hardship) or 1 (responses 3 and 4 indicating financial hardship). This question has been used previously in large population studies such as the Millennium Cohort Study (UK) (2007) and the Longitudinal Study of Australian Children (LSAC Project Operations Team 2002). Financial hardship was assessed at baseline.

### 2.4 Statistical Analysis

All analyses were conducted using statistical software, R version 4.2.0 (2022‐04‐22) with packages: Lavaan and LCMM. Growth Mixture Modelling (GMM) was used to assess parent psychological distress trajectories across the three time points while considering the time‐variant covariate (child behavioural and emotional problems) and time‐invariant covariates (child adaptive skills, SSTP intervention level, financial hardship, autism diagnosis and child age) (Ram and Grimm 2009). Notably, the time variable was coded as 0, 1 and 2 to generate more readily interpretable parameter estimates (Biesanz et al. 2004).

Guidance suggests a minimum sample size of 200, regardless of missing data or number of indicators (Kim 2012). The necessary sample size rises according to model complexity and the amount of missing data in the sample. Therefore, a limited, predetermined number of core predictors were included to balance this with the sample size available. GMM models reveal heterogeneity in the population by finding meaningful groups of participants that have similar response patterns (Wardenaar 2020). As it was expected that there would be variation in parents'/caregivers' responses to the intervention, demonstrated by different trajectories, a series of *k*‐class models were fitted (*k* = 1, …, 4).

There are two components of the analysis model to enable parameter estimates for class membership and within‐class trajectory predictors. The initial component of class membership uses a multinomial logistic model; the model allocates a probability of membership to each class (Proust‐Lima et al. 2017). An individual can only be allocated to one latent class; latent class membership is determined using the categorical variable, and the group membership can be conditional on some covariates. The second component of the model details the predictors of within‐class trajectories for the classes as mean trajectories according to time and covariates (van der Nest et al. 2020). A standard linear mixed model allows for both fixed effects and the distribution of the random effects to be class‐specific.

For the outcome variable at the baseline data collection, 1.6% of responses were missing and this increased to 23.8%, 3 months post‐intervention. This was the highest rate among all variables and time points. At the 12‐month follow‐up, 18.1% of responses for the outcome variable were missing. A Little's Missing Completely At Random (MCAR) test determined that the data were MCAR (*χ* ^2^ (371, *N* = 365) = 407, *p* = 0.0936). Therefore, further data imputation was not necessary.

The psychological distress variable was skewed and contained a number of zero scores. Therefore, the skew was removed by applying a small positive shift in the distribution (adding 1 to every value) before applying a log transformation, avoiding issues of log transformation of zero values (log[0] is undefined). Log transformations ensured that residuals were approximately normally distributed as required to reduce the possible introduction of bias in the estimates (Osborne 2002).

## 3 Results

On average, parents/caregivers reported a significant improvement in psychological distress from pre‐intervention to the 12‐month follow‐up (*t*(295) = 3.39, *p* = 0.001). On average, parents/caregivers reported a significant improvement in child behavioural and emotional problems from pre‐intervention to the 12‐month follow‐up (*t*(302) = 5.48, *p ≤* 0.001).

A baseline model was fitted looking at change across time; no other predictors were included. By examining the models across time only, it was established that change was occurring. The results suggested a three‐class model was the best fit according to the lowest AIC and BIC values (Table S1; Nylund‐Gibson et al. 2007).

Predictors of class‐membership: Child behaviour and emotional problems, child adaptive behaviour, family financial hardship, autism diagnosis and child age were then added to the models; the output is displayed in Table S2. Additional fit indexes were included to establish the best model. Both the SABIC and ICL favoured the three‐class model; therefore, it was concluded that the three‐class model continued to be the best fit.

Next, within‐class predictors were added to the model: Child behavioural and emotional problems, adaptive skills and SSTP intervention level (see Table S3). The BIC favoured a three‐class model and the three‐ and four‐class models had very similar discriminatory power. The ICL was also lowest for the three‐class model in comparison to the four‐class model. Therefore, the three‐class model was considered the best and most parsimonious fit. Class membership probability was between 0.84 and 0.97, highlighting the high likelihood of these classes being distinct.

Table 2 displays the parameter estimates for the three‐class model. The total sample size was reduced by 36 participants due to missing data. Class 1 contained 24 parents/caregivers, and these parents/caregivers had a 0.84 probability of being assigned to this class. Results are displayed in terms of the log values of the original outcome. In this class, the intercept *β* = 1.15, *p* = 0.23 means this group of parents/caregivers displayed the lowest level of psychological distress in comparison to the other groups. This group also displayed a marginal, but not statistically significant, increase in psychological distress scores over time portrayed by the slope (0.25, *p* = 0.29). Exponentiating these results ensures that interpretation is with reference to the original psychological distress scale. This group on average reported a low psychological distress score of 3 (exp(1.15)) pre‐intervention. This group was labelled as the ‘No Change’ group with a nonsignificant increase in psychological distress over time.

**TABLE 2:** Parameter estimates for the chosen three‐class model.

	Class 1	Class 2	Class 3
Sample size	24	286	19
Probability of class membership	0.84	0.97	0.91
**Estimated coefficients**			
Intercept	1.15	3.4***	2.69
Intercept variance	0.16	0.16	0.16
Slope	0.25	−0.06*	−1.35***
Slope variance	0.00	0.00	0.00
Slope–intercept covariance	0.02	0.02	0.02
Residual variance	0.56	0.56	0.56

*Note:* ***p < 0.001, *p < 0.05.

Class 2 contained the majority of parents/caregivers in the sample (*N* = 286), and these participants had a 0.97 probability of being assigned to this class. The intercept (*β* = 3.4, *p* ≤ 0.001) means this group displayed the highest level of psychological distress scores initially in comparison to the other two groups. Further, the slope (−0.06, *p* = 0.05) portrays a significant decline in psychological stress scores over time. Interpreting the intercept on the original scale, this group on average scored 30. This group was labelled the ‘Slow Improvement’ group as parents/caregivers in this group reported a significant decline in psychological distress over time.

Class 3 was composed of 19 parents/caregivers, these individuals had a 0.91 probability of being assigned to this class. Class 3 presented an intercept of *β* = 2.69, *p =* 0.64, placing this group between Class 1 and 2. This class had a slope of −1.35, *p* ≤ 0.001, indicating a significant decline over time and the steepest across the three groups. Reporting these levels based on the original measure, this group on average initially reported scores of 15 (exp(2.7)) pre‐intervention, thus half the score of Class 2 and still within the normal range for the DASS‐21 (Henry and Crawford 2005; Lovibond and Lovibond 1995). Overall, the parameter estimates for Class 3 portray the ‘Rapid Improvement’ group, as this group show a steep, significant decline in psychological distress over time.

Analysis of initial class membership is displayed in Table 3, the coefficients indicate the effect of a specific predictor on being in class ‘X’ in comparison to the reference class (Class 3) (Connell and Frye 2006). Results suggest that none of the predictors included in this part of the analysis significantly predicted class membership.

**TABLE 3:** Class membership for the three‐class growth mixture model.

	Class 1 coefficient (SE)	Class 2 coefficient (SE)
Intercept	3.03 (3.87)	2.53 (2.3)
Child behavioural and emotional problems (DBC)	1.28 (1.66)	2.25 (1.23)
Adaptive behaviour (Vineland)	−0.06 (0.05)	0.00 (0.03)
Financial hardship	0.3 (0.88)	0.35 (0.63)
Child autism diagnosis	0.2 (0.92)	−0.4 (0.64)
Child age	−0.01 (0.15)	−0.21 (0.11)

Abbreviation: SE = standard error.

Results in Table 4 display within‐class predictors for the three‐class growth mixture model, that is, the predictors that influence change in the trajectory for each class. Within‐class effects of child behavioural and emotional problems (DBC) were present for Class 1 (No Change) and Class 2 (Slow Improvement), highlighting that decreases in child behavioural and emotional problems were associated with decreases in psychological distress over time. Interpreting these findings with psychological distress on its original scale suggests that in the No Change group (Class 1), there is an exp(4.1) = 60‐point increase for every 1 point difference in DBC. The large point increase is attributable to how the DBC was scored with MIS whereby scores ranged from 0 to 2. In the Slow Improvement group (Class 2) there is an exp(0.98) = 3‐point increase for every 1 point difference in DBC. As this large change in the DBC only results in a 3‐point increase in psychological distress, this increase is not particularly meaningful.

**TABLE 4:** Within‐class predictors for the three‐class growth mixture model.

	Class 1 coefficient (SE)	Class 2 coefficient (SE)	Class 3 coefficient (SE)
Intercept	1.15 (0.95)	3.4 (0.26)***	2.69 (1.93)
Child behavioural and emotional problems (DBC)	4.1 (0.47)***	0.98 (0.11)***	−0.15 (1.14)
Adaptive behaviour (Vineland)	−0.04 (0.01)*	−0.01 (0.00)**	−0.00 (0.02)
SSTP intervention level			
Level 2	−0.24 (0.40)	−0.15 (0.12)	0.14 (0.43)
Level 3	−0.45 (0.63)	−0.06 (0.15)	−2.48 (0.56)***

*Note:* ***p < 0.001, **p < 0.01, *p < 0.05.

Abbreviation: SE = standard error.

**FIGURE 1:**
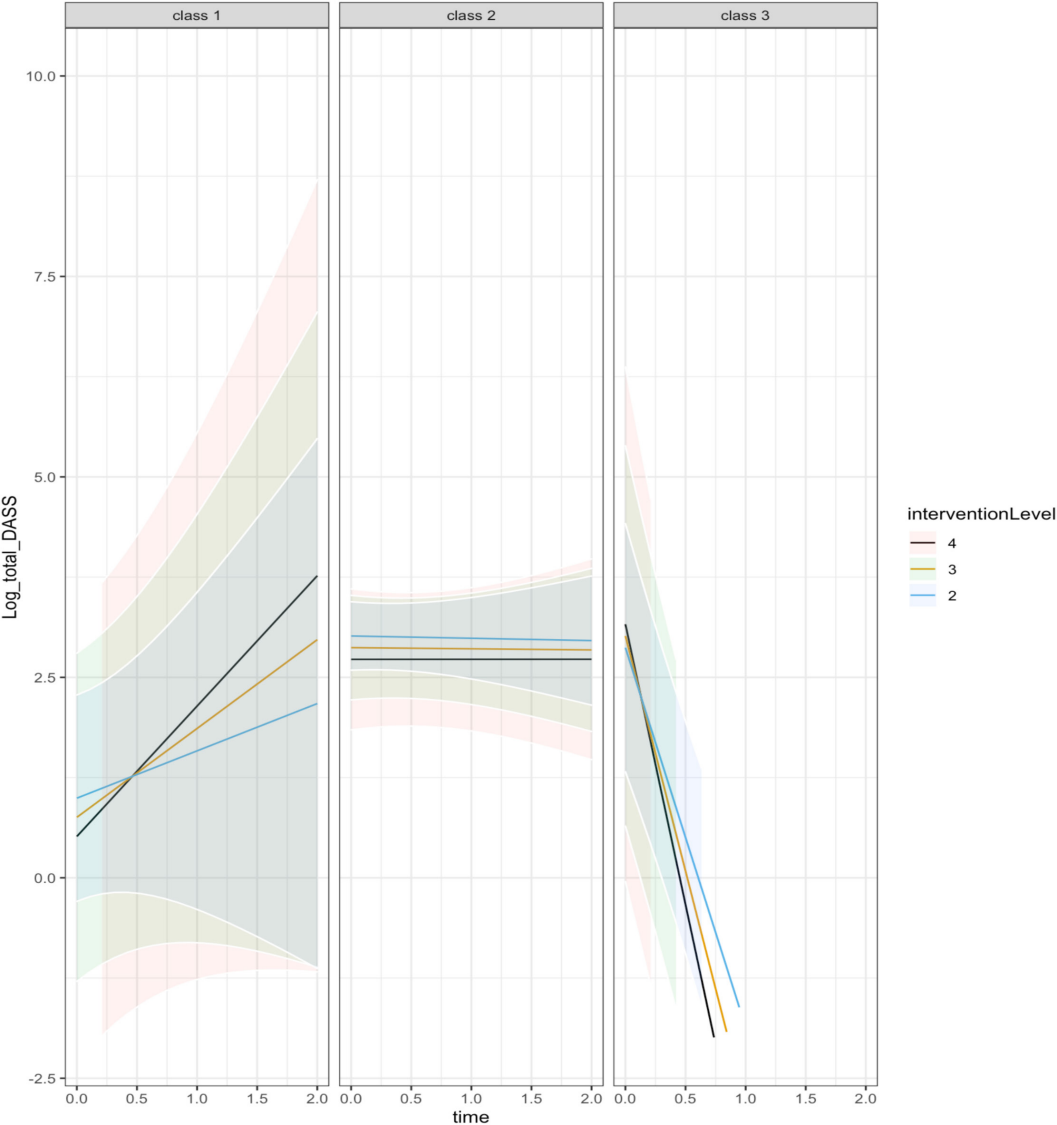
Predicted trajectories of parental psychological distress with predictors fixed at specific levels highlighting the influence of each intervention level.

Adaptive skills effects were present in the No Change group (Class 1) and the Slow Improvement group (Class 2), indicating that higher adaptive skills were related to lower psychological distress scores. When interpreting psychological distress on its original scale, in the No Change group (Class 1), for every 1 unit increase in the Vineland ABC there was an average decrease of 1 (exp(−0.04)) in psychological distress scores. In the Slow Improvement group (Class 2), for every 1 unit increase in the Vineland ABC, there was also an average exp(−0.01) = 1 point decrease in psychological distress scores. Table 4 also shows a comparison with SSTP levels whereby Level 4 (Standard SSTP) is the reference group. An effect of the SSTP intervention level was present for the Rapid Improvement group (Class 3) (*β* = −2.48, *p ≤* 0.001), the negative association indicates that Level 3 of SSTP was related to decreases in psychological distress over time. Table 4 describes a larger improvement between levels 3 and 4 (−2.48) of the SSTP intervention than between levels 2 and 4 (0.14) (see Figure 1).

The interaction between SSTP intervention level and time was then considered to observe the change over time according to SSTP intervention level. Table 5 displays the intervention level and time interaction with Level 4 as the reference group. The significant interaction in Class 3 (Rapid Improvement) (*β* = 1.38, *p ≤* 0.001), shows that psychological distress increases for Level 3 of the SSTP intervention in comparison to Level 4, for each additional unit of time. Therefore, Level 4 sees the greatest decline in psychological distress for Class 3 (Rapid Improvement) over time (see Figure 1).

**TABLE 5:** SSTP intervention and time interaction (Level 4 reference).

	Class 1 coefficient (SE)	Class 2 coefficient (SE)	Class 3 coefficient (SE)
Intercept SSTP intervention level	1.15 (0.95)	3.4 (0.26)***	2.69 (1.93)
Level 2	0.27 (0.3)	0.08 (0.07)	0.22 (0.33)
Level 3	0.18 (0.44)	−0.02 (0.09)	1.38 (0.41)***

*Note:* ***p < 0.001.

Abbreviation: SE = standard error.

To illustrate the effect of the SSTP intervention levels, Figure 1 presents the predicted psychological distress trajectories of parents/caregivers, highlighting the separation of the three classes. Log values of psychological distress are displayed so results cannot be directly related to DASS‐21 total scores. To permit plotting, all continuous predictors were fixed at specific value(s). In Class 1 (No Change), there was an apparent increase in psychological distress over time; however, as the estimates are not significant, this increase over time may be due to the level of uncertainty in the estimates, depicted by the wide confidence intervals. Figure 1 presents the small decline in psychological distress over time for Class 2 (Slow Improvement), the steep decline in Class 3 (Rapid Improvement) and the effect of intervention level for these groups; in Class 3 (High Improvement), Level 4 was associated with a steeper decline in psychological distress scores.

## 4 Discussion

This study observed three subgroups of parents/caregivers of children with developmental disabilities that reported similar levels of psychological distress over time. All groups reported psychological distress levels pre‐intervention that were within the normal range according to the DASS‐21. However, mean scores across all three time points were higher than DASS‐21 total scores from the Australian general adult population (Crawford et al. 2011). This result is consistent with findings from Gallagher et al. (2008) and Scherer et al. (2019) that parents of children with intellectual and developmental disabilities report higher levels of psychological distress. Both the Slow Improvement (Class 2) and Rapid Improvement (Class 3) groups reported a decline in the levels of psychological distress over time. The difference in improvement speed between Class 2 and Class 3 may be attributable to the difference in the baseline psychological distress levels, whereby higher levels of psychological distress relate to slower improvement rates. The No Change group (Class 1) may not have reported any improvement because initial reports of psychological distress were low and suggested no need for improvement. It is possible that parents in this group had more protective factors; for example, a higher sense of coherence has been found to be an important factor in understanding the variance in well‐being in parents of children with intellectual disabilities (Olsson and Hwang 2008). However, further research would be needed to explain the differences between groups in this sample.

Declines in psychological distress complement previous findings from SSTP research reporting reductions in levels of stress (Schrott et al. 2019) and depression (Shapiro et al. 2014). However, the absence of a control group in this study limits the conclusions that can be drawn, as psychological distress improvements may be unrelated to the intervention.

Consistent with findings from Gray et al. (2011), child behavioural and emotional problems and adaptive behaviour were associated with within‐class trajectories. In line with findings from Webster et al. (2008), higher levels of a child's adaptive skills were associated with lower levels of psychological distress over time. In the Rapid Improvement group (Class 3), SSTP intervention level was associated with the trajectory over time, with Level 4 indicating the steepest level of decline in psychological distress. This finding is consistent with evidence from Ruane and Carr (2019) that Level 4 produces more significant treatment effects. This implies that the more intense the level of SSTP is, the more likely it is to result in improvements in psychological distress over time.

As parents/caregivers were able to choose which intervention level they wanted to complete, the distribution of parents/caregivers into intervention levels is imbalanced. This, combined with the sample size of 19 from the Rapid Improvement group (Class 3), may have produced an effect that may not be present in larger, more equally distributed groups. Therefore, this may explain why the SSTP intervention level only significantly influenced the class trajectories of the Rapid Improvement group (Class 3).

None of the predictors included in this study were significantly associated with class membership; therefore, other factors not considered in this study may be influential. Despite nearly half of the sample reporting experiencing financial hardship, the majority (92.7%) of participants reported a decline in psychological distress over time. This encourages further investigation due to the implication that the SSTP programme may be associated with benefits to parents/caregivers no matter their experience of financial hardship. As child behavioural and emotional problems were significantly associated with parent/caregiver psychological distress, but an autism diagnosis was not, this is consistent with evidence from Herring et al. (2006) that child behavioural and emotional problems were a more important factor in parental mental health over time than a child's autism diagnosis. This implies that an autism diagnosis may not be a significant factor in parent/caregiver psychological distress but child behavioural and emotional problems are. The finding that a child's age was not significantly associated with parent/caregiver psychological distress differs from findings from D. Gray (2002) and Tehee et al. (2009). However, these were not intervention studies and only contained 35 and 42 parents respectively, which may explain the different findings.

### 4.1 Limitations

GGMs are restricted and exploratory, what can be interpreted from these models is constrained by what is entered and looked for (Ram and Grimm 2009). Despite meeting recommendations of the group size criterion, the smaller sample size in two of the three groups indicates a greater level of uncertainty in our estimates (Jung and Wickrama 2008). Replication with a larger sample may yield differing results due to this data‐driven approach. With a larger sample, additional predictor variables could be considered. This study was also limited to linear trajectory shapes; data on more than three occasions would be required to produce more complex nonlinear trajectories (Collins 2006).

Some parents/caregivers completed an additional intervention level between post‐intervention data collection and the 12‐month follow‐up. Consequently, this may account for changes in psychological distress during this time. Data concerning participation in a SSTP intervention during this time was not reliably collected and therefore excluded. Additionally, measures were completed predominantly by biological, adoptive, foster or step mothers (88.8%); this is a common trend in developmental disability research resulting in a lack of insight into the fathers' experience of raising a child with a developmental disability (Volker and Gibson 2014). Further, it would be beneficial in future research to obtain confirmation of an autism diagnosis from external assessments.

### 4.2 Implications

The finding that there are distinctive groups of parents/caregivers based on psychological distress and that initial levels of psychological distress may influence rates of improvement over the course of a parental intervention could be used to adapt interventions to target specific sub‐groups of parents/caregivers with further research. The importance of child behavioural and emotional problems in parental psychological distress further encourages the provision of parent programmes that aim to reduce child behavioural and emotional problems. Additional research into psychological distress trajectories is required, with data collected on more than three occasions with a larger sample to investigate if similar trends would be identified with more complex nonlinear trajectories and additional predictor variables.

## Ethics Statement

Ethical approval for the MHYPeDD study was obtained from Monash University Human Research Ethics Committee, the University of Sydney and the University of Queensland. Ethical approval for this secondary data analysis was obtained from the University of Warwick.

## Conflicts of Interest

The Triple P—Positive Parenting Program is developed and owned by the University of Queensland. Royalties from published Triple P resources are distributed to the Parenting and Family Support Centre, School of Psychology and Faculty of Health and Behavioural Sciences at the University of Queensland and contributory authors. Triple P International Pty Ltd is a private company licensed by UniQuest Pty Ltd to publish and disseminate Triple P programmes. No author has any share or ownership in Triple P International Pty Ltd. M.R. Sanders is the founder and an author on various Triple P programmes and a consultant to Triple P International. M.R. Sanders and K. Sofronoff have received, receive or may in the future receive royalties and/or consultancy fees from Triple P International. B.J. Tonge, S.L. Einfeld and K.M. Gray are authors of the Developmental Behaviour Checklist (DBC2) and related materials. All royalties received by the authors from the sale of the DBC2 materials are donated to the funding of ongoing research in intellectual and developmental disabilities.

## Supporting information


**Table S1:** Fit statistics for baseline growth mixture models with time as the only predictor for all class models.

**Table S2:** Fit statistics for growth mixture models with class‐specific predictors.

**Table S3:** Fit statistics for growth mixture models with class‐specific and within‐class predictors.

## Data Availability

The participants of this study did not give written consent for their data to be shared publicly, so due to the sensitive nature of the research, supporting data are not available.
