# Supplementary material for: Psychological Distress Trajectories of Parents of Children With Developmental Disabilities Participating in a Parenting Intervention
Source: J Intellect Disabil Res. 2025 Sep 11;69(10):1051–60. doi: 10.1111/jir.70037 (PMC12576371; doi:10.1111/jir.70037)
Supplement: Supplementary file 1 — Table S1: Fit statistics for baseline growth mixture models with time as the only predictor for all class models. [file JIR-69-1051-s003.docx]

**Supporting Information**

| Table S1. Fit statistics for baseline growth mixture models with time as the only predictor for all class models. | | | | | | | | |
| --- | --- | --- | --- | --- | --- | --- | --- | --- |
| G | loglik | npm | AIC | BIC | %class1 | %class2 | %class3 | %class4 |
| Gmm1_2 | -1240.89 | 6 | 2493.77 | 2517.12 | 100 |  |  |  |
| Gmm2_2 | - 1198.03 | 10 | 2416.07 | 2454.99 | 77.62 | 22.38 |  |  |
| Gmm3_2 | -1171.76 | 14 | 2371.52 | 2426 | 87.29 | 7.73 | 4.97 |  |
| Gmm4_2 | -1171.99 | 18 | 2379.99 | 2450.04 | 39.23 | 38.12 | 13.54 | 9.12 |
| *Note.* loglik = Log-Likelihood; npm = Number of Parameters; AIC = Akaike information criterion; BIC = Bayesian information criterion | | | | | | | | |
