# Supplementary material for: Psychological Distress Trajectories of Parents of Children With Developmental Disabilities Participating in a Parenting Intervention
Source: J Intellect Disabil Res. 2025 Sep 11;69(10):1051–60. doi: 10.1111/jir.70037 (PMC12576371; doi:10.1111/jir.70037)
Supplement: Supplementary file 2 — Table S2: Fit statistics for growth mixture models with class‐specific predictors. [file JIR-69-1051-s001.docx]

| Table S2. Fit statistics for growth mixture models with class-specific predictors. | | | | | | | | | | | |
| --- | --- | --- | --- | --- | --- | --- | --- | --- | --- | --- | --- |
| G | loglik | npm | AIC | BIC | %class1 | %class2 | %class3 | %class4 | SABIC | Entropy | ICL |
| Gmm2_2CP | -1066.81 | 15 | 2163.61 | 2220.56 | 64.44 | 35.56 |  |  | 2172.98 | 0.58 | 1647.01 |
| Gmm3_2CP | -1041.96 | 24 | 2131.91 | 2223.01 | 4.26 | 70.21 | 25.53 |  | 2146.87 | 0.75 | 1642.41 |
| Gmm4_2CP | -1032.50 | 33 | 2131.01 | 2256.28 | 37.69 | 43.77 | 18.24 | 0.30 | 2151.60 | 0.78 | 1680.16 |
| *Note.* loglik = Log-Likelihood; npm = Number of Parameters; AIC = Akaike information criterion; BIC = Bayesian information criterion; SABIC= Sample-Size Adjusted BIC, lower scores indicate better fit; Entropy values indicate levels of discriminatory power with values ranging from 0.00 – 1.00; ICL = Integrated Complete Likelihood, an adaptation of the AIC and BIC indexes that adjusts for entropy, a lower value indicates better fit. | | | | | | | | | | | |
