# Supplementary material for: Psychological Distress Trajectories of Parents of Children With Developmental Disabilities Participating in a Parenting Intervention
Source: J Intellect Disabil Res. 2025 Sep 11;69(10):1051–60. doi: 10.1111/jir.70037 (PMC12576371; doi:10.1111/jir.70037)
Supplement: Supplementary file 3 — Table S3: Fit statistics for growth mixture models with class‐specific and within‐class predictors. [file JIR-69-1051-s002.docx]

| Table S3. Fit statistics for growth mixture models with class-specific and within-class predictors. | | | | | | | | | | | |  |
| --- | --- | --- | --- | --- | --- | --- | --- | --- | --- | --- | --- | --- |
| G | loglik | npm | AIC | BIC | %class1 | %class2 | %class3 | %class4 | SABIC | Entropy | ICL |  |
| Gmm1_2WCP | -1088.06 | 10 | 2200.12 | 2245.82 | 100 |  |  |  | 2207.75 | 1 | 2245.82 |  |
| Gmm2_2WCP | -1027.62 | 26 | 2107.24 | 2205.94 | 90.27 | 9.73 |  |  | 2123.47 | 0.9 | 1564.14 |  |
| Gmm3_2WCP | -1000.36 | 40 | 2080.71 | 2232.55 | 7.3 | 86.93 | 5.78 |  | 2105.67 | 0.88 | 1605.06 | |
| Gmm4_2WCP | -977.029 | 54 | 2062.06 | 2267.05 | 5.76 | 62.61 | 23.40 | 8.21 | 2095.76 | 0.91 | 1637.96 |  |
| *Note.* loglik = Log-Likelihood; npm = Number of Parameters; AIC = Akaike information criterion; BIC = Bayesian information criterion; SABIC= Sample-Size Adjusted BIC, lower scores indicate better fit; Entropy: levels of discriminatory power (0.00 – 1.00); ICL = Integrated Complete Likelihood, an adaptation of the AIC and BIC indexes that adjusts for entropy, a lower value indicates better fit. | | | | | | | | | | | |  |
